# Model-based optimization approaches for precision medicine: A case study in presynaptic dopamine overactivity

**DOI:** 10.1371/journal.pone.0179575

**Published:** 2017-06-14

**Authors:** Kai-Cheng Hsu, Feng-Sheng Wang

**Affiliations:** 1 Department of Neurology, National Taiwan University Hospital Yunlin Branch, Yunlin, Taiwan; 2 Department of Chemical Engineering, National Chung Cheng University, Chiayi, Taiwan; Istituto di Genetica Molecolare, ITALY

## Abstract

Precision medicine considers an individual’s unique physiological characteristics as strongly influential in disease vulnerability and in response to specific therapies. Predicting an individual’s susceptibility to developing an illness, making an accurate diagnosis, maximizing therapeutic effects, and minimizing adverse effects for treatment are essential in precision medicine. We introduced model-based precision medicine optimization approaches, including pathogenesis, biomarker detection, and drug target discovery, for treating presynaptic dopamine overactivity. Three classes of one-hit and two-hit enzyme defects were detected as the causes of disease states by the optimization approach of pathogenesis. The cluster analysis and support vector machine was used to detect optimal biomarkers in order to discriminate the accurate etiology from three classes of disease states. Finally, the fuzzy decision-making method was employed to discover common and specific drug targets for each classified disease state. We observed that more accurate diagnoses achieved higher satisfaction grades and dosed fewer enzyme targets to treat the disease. Furthermore, satisfaction grades for common drugs were lower than for specific ones, but common drugs could simultaneously treat several disease states that had different etiologies.

## Introduction

The concept of precision medicine has been recognized not only by clinicians and biomedical researchers but also by patients and policy makers in recent years [1]. Precision medicine is defined as treatments tailored to individual patients on the basis of their genetic, biomarker, phenotypic, and psychosocial characteristics that distinguish a given patient from other patients with similar clinical presentations [2]. The goals of precision medicine are to identify an individual’s susceptibility to disease, obtain an accurate diagnosis, and deliver an efficient treatment. Although some successful examples of precision medicine are present in oncology, relatively few examples exist in psychiatry [3, 4].

Schizophrenia is a chronic psychiatric disorder that afflicts approximately 1% of the population worldwide, but its cause remains unknown [5]. Evidence of dopamine (DA) involvement in schizophrenia developed with the approval of the antipsychotic drug Reserpine in 1954, which was reported to block the accumulation of DA into secretory vesicles [6]. Other first-, second-, and third-generation antipsychotic drugs have been found to correlate with their binding affinities for D2-type DA receptors [7–10]. Furthermore, several imaging studies using positron emission tomography have shown presynaptic dopaminergic dysfunction with dopamine overactivity in schizophrenia [11–14].

The current diagnosis of psychiatric disorders, including schizophrenia, is mainly based on a clinician’s assessment according to the Diagnostic and Statistical Manual of Mental Disorders, 4th Edition (DSM-IV) and International Statistical Classification of Diseases and Related Health Problems (ICD-10) diagnostic criteria. However, different disorders often manifest similarly, and the overlap of symptoms results in diagnostic ambiguity [15]. Diagnostic biomarkers using imaging techniques [16] and molecular biology techniques [17] may increase the accuracy of diagnostic strategies and allow disease classifications to be more effectively targeted for personalized therapy. One molecular biomarker alone may not possess a strong enough statistical power to predict disease state, especially with complex diseases. Additionally, these diseases not only require more accurate diagnostic tools but also need to be viewed more thoroughly in terms of their dynamic behaviors. Therefore, a set of biomarkers identified through systems biology approaches can be used as a panel to characterize a disease [18].

In contrast to the trial and error method, mathematical modeling and optimization are emerging technologies for drug discovery for human metabolic disorders [19–23]. In our previous studies [24, 25], we have introduced a fuzzy multiobjective optimization process for formulating the enzyme target design problem to identify drug targets. Prevention and treatment strategies for diseases should consider individual variability in precision medicine [1]. Therefore, we introduced a model-based optimization approach in this study to identify the pathogenesis of presynaptic dopamine overactivity, not only to determine a one-hit enzyme defect and two-hit enzyme defects but also their corresponding dysregulated level. Furthermore, such disorder observations were used to identify the candidates of biomarkers in order to classify different etiologies of presynaptic dopamine overactivity. Finally, a fuzzy decision-making approach was introduced to discover drug targets for various disease cases; that is, common targets for several disease states because of their different etiologies and specific targets for each disease state because of their specific etiologies were considered.

## Method

**Fig 1:**
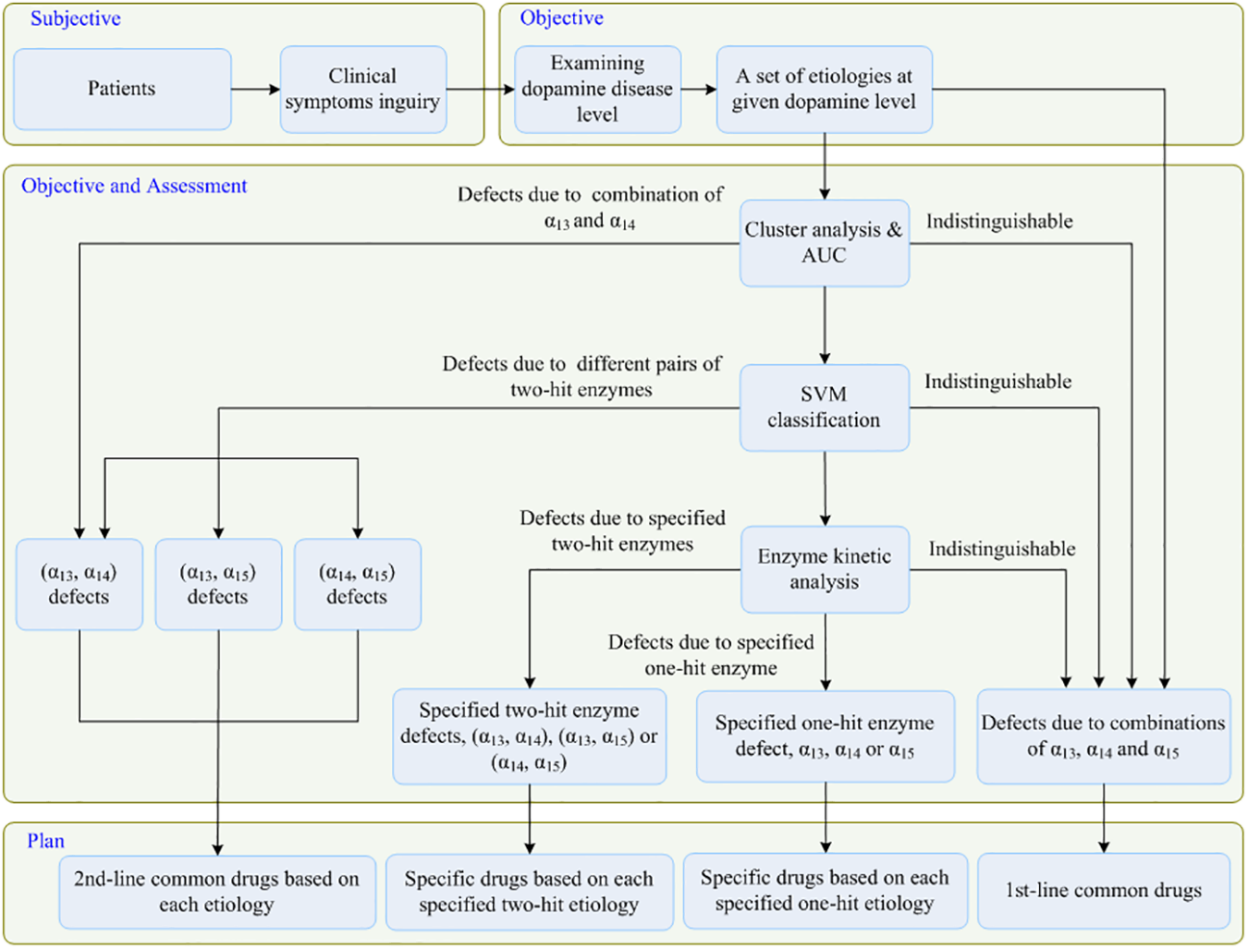
Roadmap of model-based optimization for precision medicine. Subjective component: The symptoms are inquired about according to DSM-IV and ICP-10 diagnostic criteria for the diagnosis of possible presynaptic dopamine overactivity. Objective component and assessment: Dopamine level is measured by image studies. Then, a single biomarker must be tested for identifying Class 1 patients with α_13_ and α_14_ defects. Furthermore, three to four biomarkers are examined for identifying Class 2 or Class 3 patients with α_13_ and α_15_ or α_14_ and α_15_ defects, respectively. Finally, if the enzyme activity can be tested, the specific disease states with precise single or multiple enzyme defects are obtained. Plan: When the dopamine level is determined, the 1st-line common drugs can be used without further information concerning enzyme defects. The 1st-line common drugs are compatible with current medications because tests for enzyme defects are not clinically available. After single or multiple biomarkers are examined to identify Class 1, 2, and 3 patients, 2nd-line common drugs are utilized. The 2nd-line common drugs are more effective than are the 1st-line common drugs. When specific enzyme defects are detected, the specific drugs can be discovered to treat patients with the highest therapeutic effect, the lowest adverse effect, and the lowest drug dose. Hence, the specific drugs are indicated for use in patients who are refractory to 1st- and 2nd-line common drugs.

This study proposes the model-based optimization approach for precision medicine for treating a patient with presynaptic dopamine overactivity as shown in Fig 1. The procedures are consisted of three parts, namely detection of pathogenesis, biomarker identification, and drug target discovery, and are similar to SOAP notes [26], which document subjective (S), objective (O), assessment (A), and plan (P) details, are a highly structured format for documenting the progress of a patient during treatment. Such notes are part of the problem-oriented medical record approach most commonly used by physicians and other healthcare professionals. The proposed computations are generally developed in advance to provide as a medical aided systems. Each computational procedure is explained as following.

### Detection of pathogenesis

**Fig 2:**
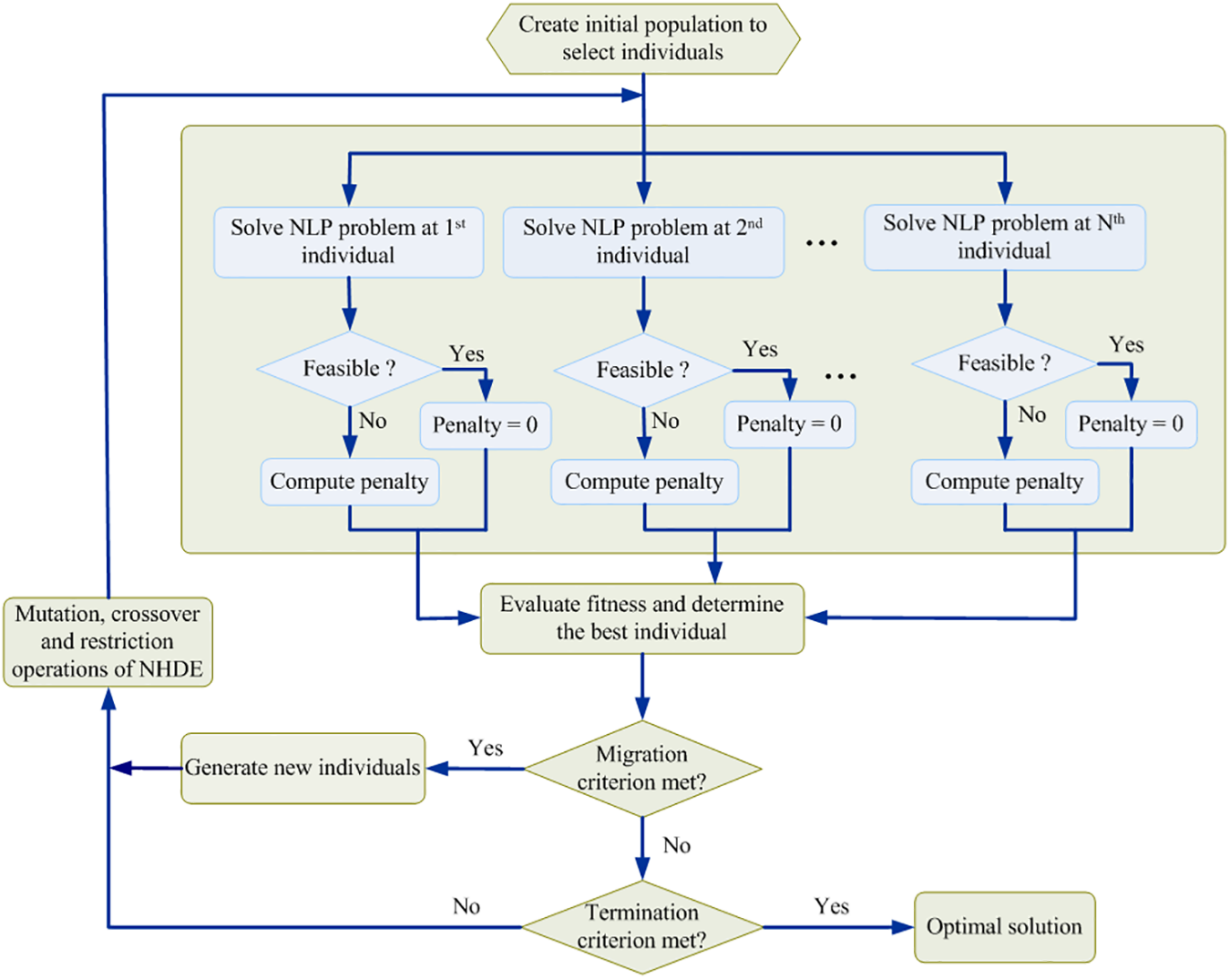
Flowchart for the modified algorithm for NHDE. The core procedure of the NHDE algorithm is the evaluation and selection operation as shown in the second and third block diagram of the flowchart. The evaluation step is to solve each nonlinear programming (NLP) problem produced from the maximizing decision problem for each target candidate. The fitness should be accompanied by a penalty value for infeasible solutions. The fitness of each NLP problem is computed for selecting the better individuals in the population, and then to generate the next individuals. The migration operation of NHDE is used to help all individuals escape from this local cluster. This migration operation is performed only if the measure of population diversity fails to satisfy the desired tolerance.

Sensitivity analysis is a conventional method for examining which enzyme is the most sensitive deficient in a network to trigger pathogenesis [27]. However, sensitivity analysis only changes each enzyme activity in a small amount to evaluate variation of the predicted metabolite concentrations, and cannot detect disease levels caused by enzyme defects. The most effective enzymes of pathogenesis can be detected through the model-based optimization problem as follows:

$$\begin{array}{l} \underset{x,\hat{\alpha },u,z}{\min}\sum_{i\in \Omega^{DS}}{\left(x_{i}-x_{i}^{disease}\right)}^{2}+\sum_{j\in \Omega^{Enz}}{\left(1-{\hat{\alpha }}_{j}/{\hat{\alpha }}_{j}^{basal}\right)}^{2}+\sum_{j\in \Omega^{EX}}{\left(1-u_{j}/u_{j}^{basal}\right)}^{2}\\ \mathrm{subjec}\;\mathrm{to}\\ \mathrm{Material}\;\mathrm{balance}\;\mathrm{equations:}\\ \left\{ \begin{array}{l} \sum_{j=1}^{r}N_{ij}v_{j}\left(x,\alpha \right)+\sum_{j=1}^{m}B_{ij}u_{j}=0,i\in \Omega^{SP}\\ w_{j}={\hat{\alpha }}_{j}+\sum_{k=1}^{n_{r}}g_{jk}y_{k},j\in \Omega^{Rxn}\\ v_{j}=\exp\left(w_{j}\right)\\ x_{i}=\exp\left(y_{i}\right)\\ \mathrm{Metabolite}\;\mathrm{constraints:}\\ x_{i}^{LB}\leq x_{i}\leq x_{i}^{UB};\;i\in \Omega^{SP} \end{array}\right.\\ \left\{ \begin{array}{l} \mathrm{Target}\;\mathrm{constraints}:\\ \mathrm{up-regulation}:\left\{ \begin{array}{l} {\hat{\alpha }}_{z}^{basal}\leq {\hat{\alpha }}_{z}\leq {\hat{\alpha }}_{z}^{UB},z\in \Omega_{z}^{up}\\ u_{z}^{basal}\leq u_{z}\leq u_{z}^{UB} \end{array}\right.\\ \mathrm{down-regulation}:\left\{ \begin{array}{l} {\hat{\alpha }}_{z}^{LB}\leq {\hat{\alpha }}_{z}\leq {\hat{\alpha }}_{z}^{basal},z\in \Omega_{z}^{down}\\ u_{z}^{LB}\leq u_{z}\leq u_{z}^{UB} \end{array}\right.\\ {\hat{\alpha }}_{z}={\hat{\alpha }}_{z}^{basal},z\notin \Omega_{z}=\Omega_{z}^{up}+\Omega_{z}^{down} \end{array}\right. \end{array}$$ (1)

where *x*_*i*_, *i* = 1,…,*n* are the metabolite concentrations, ${\hat{\alpha }}_{j},j=1,\ldots ,r$ are the logarithmic enzyme activities, *N*_*ij*_ is the stoichiometric coefficient of metabolite *i* in reaction *j*, *v*_*j*_ is the reaction rate *j*, and *B*_*ij*_ is the connectivity coefficient of metabolite *i* in external control *u*_*j*_. The formulation in Eq (1) is a mixed-integer nonlinear programming (MINLP) problem whereby a set of integer variables **z** are first detected, and their corresponding up and down regulatory levels of enzyme activities and external controls are determined to minimize the objective function. The first term of the objective function is used to account for the metabolite concentrations of the disease state as close to their disease levels, $x_{i}^{disease}$ as possible, the second and third terms are used to determine the detected enzymes and external inputs with as little variation to its basal level, ${\hat{\alpha }}_{j}^{basal}$ and $u_{j}^{basal}$, as possible. The MINLP problem is an NP-hard problem and is difficult to solve. Generally, the problem may contain multiple dysregulated inputs to yield the identical disease situation. Nested hybrid differential evolution (NHDE) can be applied to solve the NP-hard problem; NHDE iteratively found a set of pathogenic enzymes (Fig 2, S1 File).

### Biomarker identification

#### Cluster and statistical analysis

Hierarchical cluster analysis was used to classify a given dataset into homogeneous groups that differ from each other on the basis of given parameters [28]. This analysis was performed with a between-groups linkage, and the cluster distance was expressed as the square Euclidean distance [29]. A set of etiologies obtained from the pathogenesis problem (1) were clustered using a dendrogram employing the average linkage method into two groups. Between-group comparisons were made using independent-samples T tests and ANOVA tests. Receiver operating characteristic (ROC) curves were plotted and areas under curves (AUCs) were calculated. P < 0.05 was considered significant.

#### Support vector machine

Support vector machine (SVM) was applied to identify biomarkers. SVM was first introduced in 1995 [30] and has been used in many pattern recognition problems [31–33] and biological problems [33–36]. SVM determines the optimal hyperplane that has the maximum margin to classify data into two classes [34, 37–39]. The data for a two-class learning problem consists of objects labeled with one of two labels corresponding to two classes; for convenience we assume the labels y are +1 (positive patterns) or −1 (negative patterns). Let **x** denote a vector of n-dimensional components *x*_*i*_, *i* = 1,…,*n*. The object **x**_*i*_ is called a pattern in the dataset [(**x**_*i*_, y_i_), *i* = 1,…,*m*]. A linear classifier is based on a linear discriminant function of the form

$$f\left(x\right)=w^{T}x+b$$ (2)

where the vector **w** is the weight vector and the scalar *b* is the bias. Eq (2) is a hyperplane, that is, a linear decision boundary, to linearly separate the dataset into positive or negative patterns. For a given hyperplane, we can determine the maximum margin classifier that maximizes the geometric margin, which is equivalent to minimizing the soft-margin SVM problem of the form

$$\left\{ \begin{array}{l} \underset{w,b,\xi_{i}}{\min}\frac{1}{2}{\left\|w\right\|}^{2}+C\sum_{i=1}^{m_{t}}\xi_{i}\\ \mathrm{subject}\;\mathrm{to}\\ y_{i}\left(w^{T}x_{i}+b\right)\geq 1-\xi_{i},i=1,\ldots ,m_{t}\\ \xi_{i}\geq 0 \end{array}\right.$$ (3)

where *m*_*t*_ is the number of training sets. Once the optimal values of **w**, *b* and ξ_*i*_ are found, the patterns in the test set can be classified by the discriminant function *f*(**x**) either into the +1 or −1 class. The classifier of the problem (3) can be usually obtained by solving its dual problem.

SVM was originally designed for binary classification. To solve a multiclass problem, we decompose the multiclass problem into a series of two-class problems and construct several binary classifiers [30, 37–41]. The one-versus-all method, which was the earliest and one of the most widely used methods, was implemented. This method involves a parallel architecture comprising M-SVMs [41]. Each SVM solves a two-class problem defined by one information class. The “winner-takes-all” rule is used for the final decision, that is, the winning class is the one corresponding to the SVM with the highest output.

**Fig 3:**
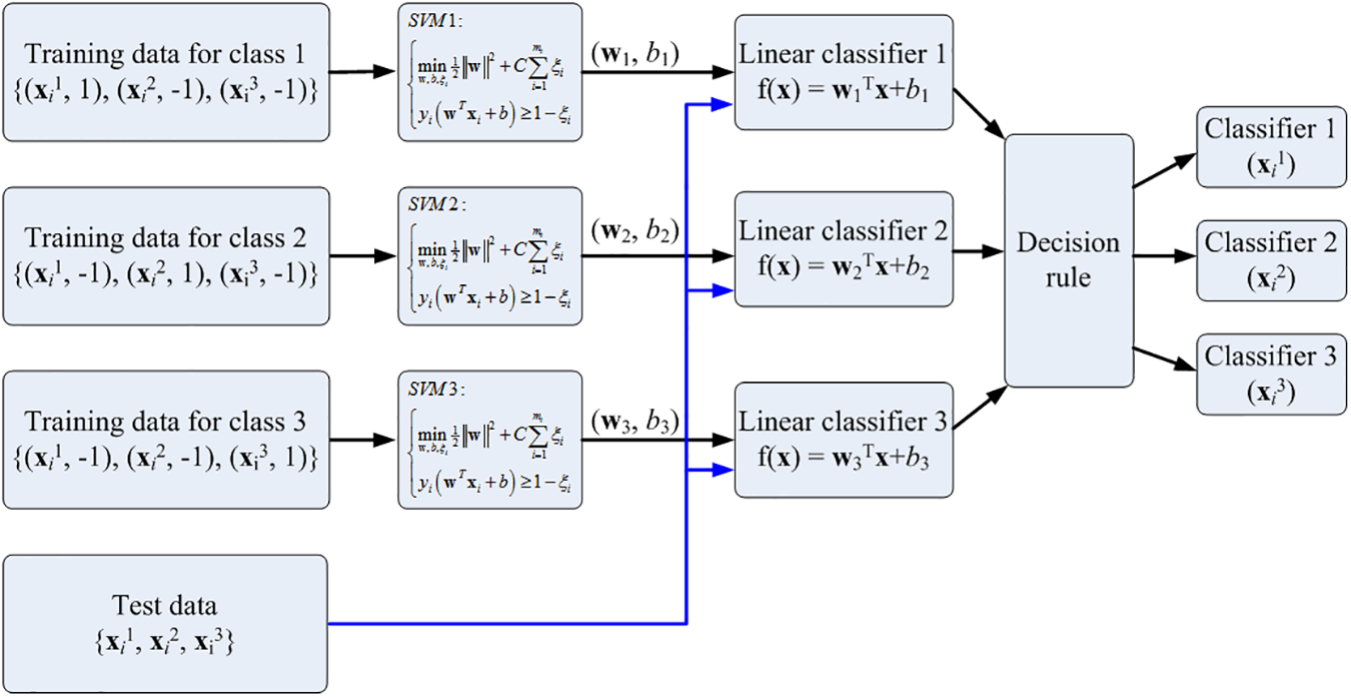
Flowchart for the triple SVM classifiers. 50% of data from class 1, 2, and 3 were used for training. A parallel architecture comprising triple SVMs was employed. The rest 50% of data were used for testing. Each SVM solves a two-class problem defined by one information class. The “winner-takes-all” rule is used for the final decision. The winning class is the one corresponding to the SVM with the highest output.

Triple SVM classifiers as shown in Fig 3 were applied to classify the three classes of data. First, 50% of the data from Classes 1 and 2 were randomly selected for the training of SVM1. Second, the trained SVM1 was used to classify the remaining 50% of the data from Classes 1, 2, and 3. Third, the SVM2 classifier trained using the training data from Class 2 and 3 was used to classify the test signals from Classes 1, 2, and 3. Fourth, the SVM3 classifier trained using the training data from Classes 1 and 3 was used to classify the test data from Classes 1, 2, and 3. Finally, the classified results associated with each pair of classes were analyzed and the final classified class was determined using the most frequently classified class. All SVM computational results were obtained using the MATLAB (version R2015a) environment by using the bioinformatics toolbox.

### Drug target discovery

This study introduced a fuzzy multiobjective optimization to discover a set of drug targets to recover disease states because of different etiologies. Hsu and Wang [25] proposed a fuzzy decision-making approach to detect enzyme targets in the presynaptic dopamine metabolic network, respectively, to return two types of dopamine deficiencies to their normal state. Such an approach is only suited for one-enzyme dysregulation. This study extended the optimization problem to detect the specific targets for recovering disease state caused to each specific enzymopathy, that is, precise medical treatment for each dysregulated case and for a common treatment. Different enzymopathies can generally cause an identical dopamine overexpressed level. The aforementioned biomarker must be applied to identify each specific enzymopathy and to discover each specific drug target. In contrast to such a personalized treatment, we were unable to completely identify each specific enzymopathy if biomarker tests were not available, so common drug targets must be used to treat multiple diseases. The fuzzy optimization formulation for treating multiple enzymopathies is expressed as follows:

$$\left\{ \begin{array}{l} \mathrm{Therapeutic}\;\mathrm{effect}:\underset{x,\hat{\alpha },u,z}{Fuzzy\;equal}\;x_{id}\approx x_{i}^{basal};\;i\in \Omega^{TH},d\in \Omega^{DS}\\ \mathrm{Adverse}\;\mathrm{effect}:\underset{x,\hat{\alpha },u,z}{Fuzzy\;\min}\;x_{jd};\;j\in \Omega^{AE},d\in \Omega^{DS}\\ \mathrm{Variation}\;\mathrm{effect}:\underset{x,\hat{\alpha },u,z}{Fuzzy\;equal}\;{\hat{\alpha }}_{z_{k}}\approx {\hat{\alpha }}_{z_{k}}^{basal};\;z_{k}\in \Omega^{TE}=\Omega^{Rxn}\backslash \Omega^{DS}\\ \underset{x,\hat{\alpha },u,z}{\;Fuzzy\;equal}\;u_{z_{k}}\approx u_{z_{k}}^{basal};\;z_{k}\in \Omega^{EX}\\ \mathrm{Number}\;\mathrm{of}\mathrm{targets}:\underset{x,\hat{\alpha },u,z}{\min}\sum_{k}z_{k};z_{k}\in \Omega^{TE}\\ \left\{ \begin{array}{l} \mathrm{Material}\;\mathrm{balance}\;\mathrm{equations}:\\ \sum_{j=1}^{r}N_{ij}v_{j}\left(x,\alpha \right)+\sum_{j=1}^{m}B_{ij}u_{j}=0;\;i\in \Omega^{SP},d\in \Omega^{DS}\\ \mathrm{Metabolite}\;\mathrm{constraints}:\\ x_{i}^{LB}\leq x_{id}\leq x_{i}^{UB} \end{array}\right.\\ \left\{ \begin{array}{l} \mathrm{Target}\;\mathrm{constraints}:\\ {\hat{\alpha }}_{z_{k}}^{LB}\leq {\hat{\alpha }}_{z_{k}}\leq {\hat{\alpha }}_{z_{k}}^{UB};\;z_{k}\in \Omega^{TE}\\ {\hat{\alpha }}_{z_{k}}={\hat{\alpha }}_{z_{k}}^{basal};\;z_{k}\notin \Omega^{TE}\\ u_{z_{k}}^{LB}\leq u_{z_{k}}\leq u_{z_{k}}^{UB};z_{k}\in \Omega^{EX}\\ u_{z_{k}}=u_{z_{k}}^{basal};z_{k}\notin \Omega^{EX} \end{array}\right. \end{array}\right.$$ (4)

The therapeutic and adverse objectives in the fuzzy optimization problem consider different disease states. However, the common drug targets—enzyme activities and external inputs—are identical for different diseases, so the variation effects are indiscriminate. The final objective is to minimize the overall design targets that consist of enzyme activities and external controls. The *i*^th^ reaction rate, *v*_*id*_, in the material balance equation, depends on each disease state and is expressed in the power law function as follows:

$$v_{jd}=\alpha_{jd}\prod_{k=1}^{n}x_{k}^{g_{jk}}$$ (5)

where the rate constant or enzyme activity, α_*jd*_, depends on whether the rate is normal or disease state, that is,

$$\alpha_{jd}=\left\{ \begin{array}{l} \alpha_{j},\;j\in \Omega^{TE}\mathrm{and}j\notin \Omega^{DS}\\ \alpha_{j}^{basal},\;j\in \Omega^{DS}\mathrm{and}j\neq d\\ \alpha_{j}^{DS},\;j\in \Omega^{DS}\mathrm{and}j=d \end{array}\right.$$ (6)

The optimal drug target discovery problem in Eq (4) is a generalized optimization problem. It is used not only for detecting a common target but also for finding a specific target if the pathogenic enzyme can be completely identified. We applied a fuzzy decision making method–elicited generalized membership function to solve the problem. This approach is extended from our previous work but must handle multiple disease states. The optimization problem (4) can be transformed into a fuzzy decision-making problem as shown in supplementary (S2 File) and can then be solved using the NHDE algorithm (Fig 2 and S1 File) to obtain the optimal drug targets for different defects.

## Results

We used a case of presynaptic dopamine overactivity, which may cause schizophrenia, as a case study to illustrate the performance of the proposed strategy in Fig 1. The mathematical model of the nigrostriatal dopamine network accessed from Qi et al. [42, 43] is expressed as the generalized mass action framework that consisted of 34 metabolites, 18 independent variables, and 68 target enzymes. The detailed definition of the model is expressed in supporting information (S3 File). Here, the extracellular dopamine (DA-e) concentration was obtained from the model at steady state to be 400 (relative unit) assigned as the healthy state (HS). Other metabolite concentrations of interest include reactive oxygen species (ROS), reactive nitrogen species (RNS), and toxic species (for HS values, see S3 File).

### Pathogenesis of presynaptic dopamine overactivity

**Fig 4:**
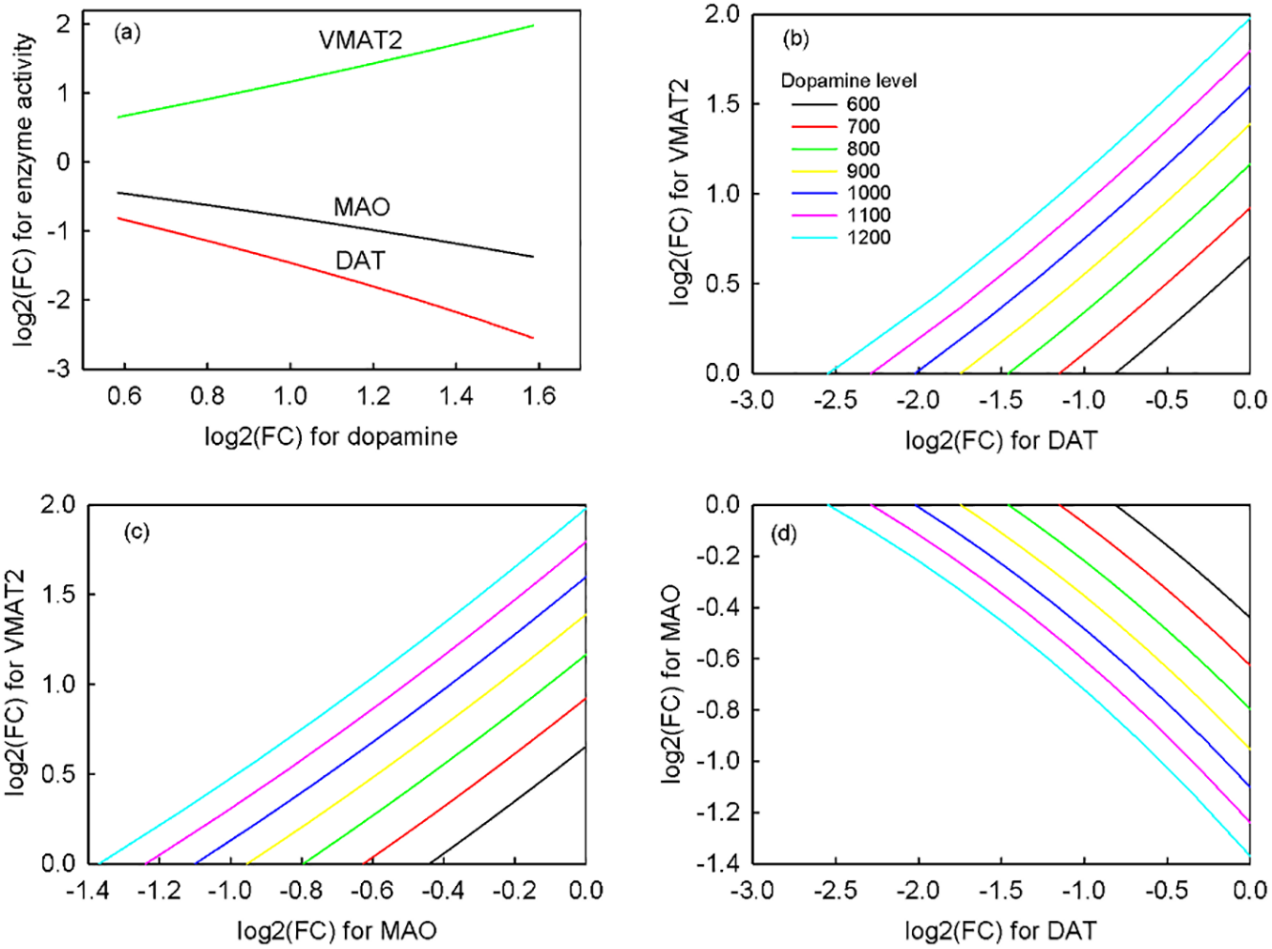
Dysregulated ratios of one-hit and two-hit enzymes. (a). single enzyme defect, (b). DAT and VMAT2 defects, (c). MAO and VMAT2 defects, (d). DAT and MAO defects. The enzyme activities of MAO and DAT decrease and the enzyme activity of VMAT2 increases. The combinations of two-enzyme dysregulation from the pairs of MAO, DAT, and VMAT2 cause an overexpression of various dopamine levels, namely 600, 700, 800, 900, 1000, 1100, and 1200. FC denotes the fold change between the disease state and the health state.

After a clinical symptoms inquiry, the initial step in the objective section of Fig 1 is to examine dopamine overactive level, and then its disease value $x_{i}^{disease}$ is provided for pathogenesis problem (1) to determine a set of etiologies. To illustrate a wider region of pathogenesis, we considered seven disease states, that is, dopamine concentrations from 600 to 1200 (relative unit). The NHDE algorithm was applied to determine the one-hit defect to trigger increasing dopamine concentrations, and we found that two-enzyme activities decreased and one-enzyme activity increased, resulting in different overexpressed levels of dopamine (Fig 4A). In dopamine of 600 (equivalent to log2(D/N) = 0.585), we detected that three enzyme activities decreased, namely monoamine oxidase (MAO) (−26.3%), dopamine transporter (DAT) (−43.1%), and catechol-O-methyltransferase (COMT) (−93%) (not shown in Fig 4A), whereas one enzyme—vesicular monoamine transporter 2 (VMAT2)—increased (+57.3%) to reach this disease level. However, we could not find a feasible result in favor of a COMT defect when the dopamine level exceeded 600. From Fig 4A, we observed that smaller enzyme defects, resulting in higher dopamine overexpression, and MAO, were a key enzyme, compared with DAT and VMAT2, which causes presynaptic dopamine overexpression. The relative sensitivity of dopamine concentration (S1 Table) with respect to each enzyme activity was also applied to evaluate the regulation effects. Downregulation of MAO and DAT and upregulation of VMAT2 could raise dopamine levels. This observation was consistent with the optimization search as shown in Fig 4A.

The two-hit hypothesis, also known as the Knudson hypothesis, is the hypothesis that two mutant alleles can initiate tumorigenesis [44]. Such a two-hit hypothesis can be applied to infer schizophrenia that multiple genetic alterations have been suggested to affect the development of the central nervous system in a manner that creates an abnormal signaling network [45]. In this work, the pathogenesis problem was also applied to detect the two-enzyme dysregulation in order to interpret the behaviors. All possible combinations of two-enzyme dysregulation were evaluated from the pairs of MAO, DAT, and VMAT2. Fig 4B, 4C and 4D show that different dysregulated pairs of two-hit defects cause an overexpression of various dopamine levels. These results were then used for cluster analysis and for SVMs to identify biomarkers and to discover the first- and second-line drug targets.

### Biomarkers detected by cluster analysis

To identify the possible biomarkers for presynaptic dopamine overactivity, we classified the disease states into different groups depending on the similarity of metabolites. We calculated the logarithmic ratio based on two of the metabolites as the form.

$$R_{i,}{}_{\log2}=\log2\left(\frac{x_{i}^{disease}}{x_{i}^{basal}}\right)$$ (7)

**Fig 5:**
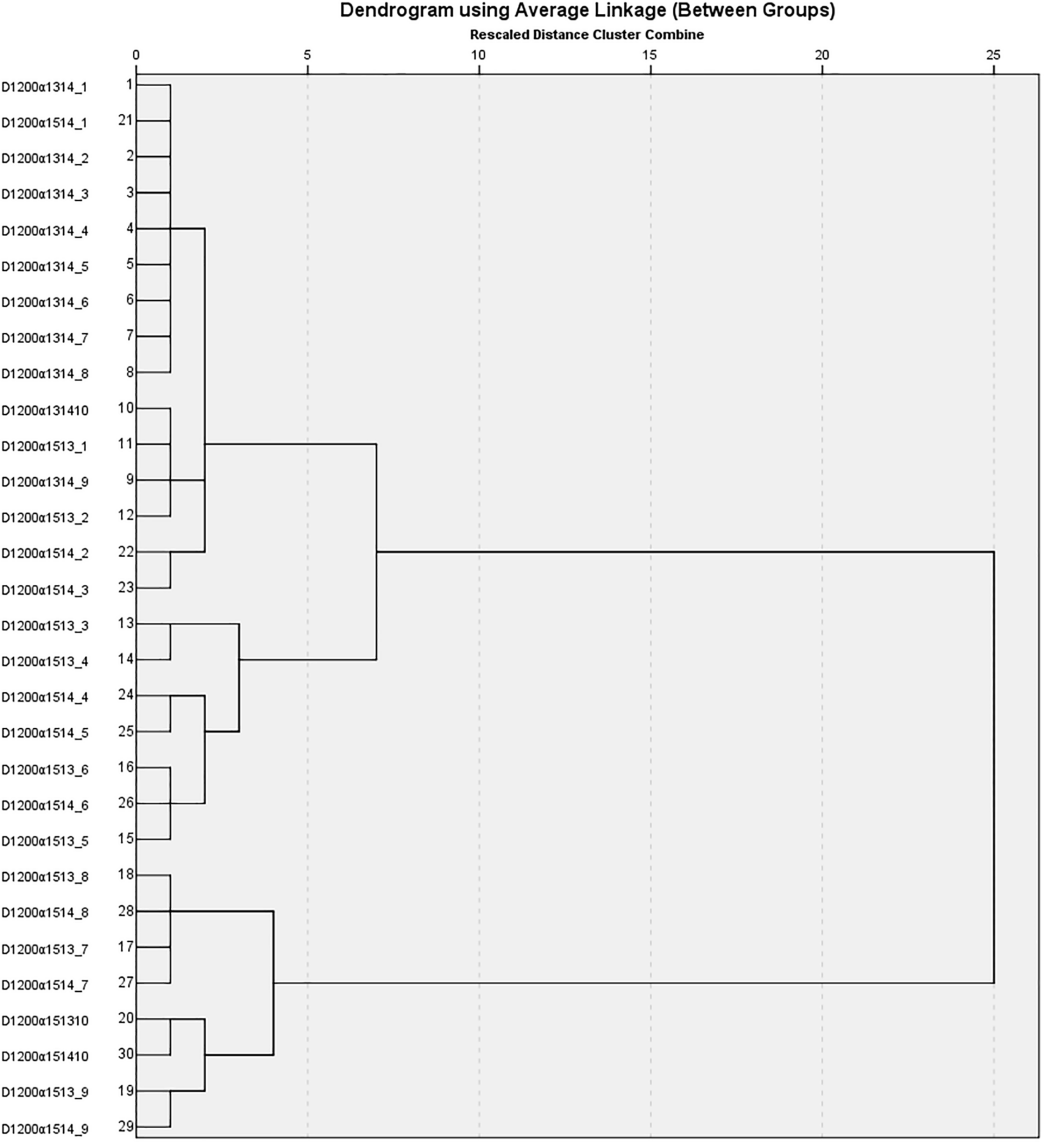
Hierarchical tree in dopamine disease level of 1200. The presynaptic dopamine overactivity with different etiologies could be qualitatively divided into two groups: one is the one with the MAO (α15) defect, and the other is without the MAO defect. D1200α1314_1 denotes dopamine disease level of 1200 with DAT (α13) and VMAT2 (α14) defects, and we use 10 different combinations of two-enzyme dysregulation for cluster analysis.

Then, hierarchical clustering was employed to classify various dopamine disease levels with different etiologies separately based on $R_{i,\log2}$. Fig 5 shows the hierarchical tree in dopamine disease level of 1200. According to the hierarchical clustering result, the presynaptic dopamine overactivity with different etiologies could be qualitatively divided into two groups: one is the one with the MAO (α15) defect, and the other is without the MAO defect. We compared the $R_{i,\log2}$ of metabolites in these two groups by applying the independent-samples T tests. The ratios of the 24 metabolites were statistically different in these two groups. Furthermore, we calculated the AUC of ROC curves (Table 1, other dopamine disease levels see S2 Table), and the values were greater than 0.95 with p < 0.001. Consequently, these 24 metabolites could be used as biomarkers to distinguish the states of presynaptic dopamine overactivity in those with a MAO defect from those without a MAO defect.

**Table 1:** Candidates for biomarkers selected by cluster analysis and ROC curves at dopamine disease level of 1200.

Metabolites	p-value	AUC
Tyrosine (*x*_1_)	<0.001	0.98
Dihydrobiopterin (*x*_2_)	<0.001	0.95
Tyramine (*x*_3_)	<0.001	0.95
L-DOPA produced from tyrosine (*x*_4_)	<0.001	0.95
Dopaquinone (*x*_5_)	<0.001	0.95
Pyrrolo-quinoline quinone (*x*_6_)	<0.001	0.95
Intracellular dopamine (*x*_7_)	<0.001	0.95
Prostaglandin H_2_ (*x*_15_)	<0.001	0.95
Dopamine quinone (*x*_16_)	<0.001	0.95
Dopamine chrome (*x*_17_)	<0.001	0.95
L-Dopachrome (*x*_19_)	<0.001	0.95
5,6-Dihydroxyindole (*x*_20_)	<0.001	0.95
Indole-5,6-quinone (*x*_21_)	<0.001	0.95
5,6-Dihydroxyindole-2-carboxylate (*x*_22_)	<0.001	0.95
Melanin (*x*_23_)	<0.001	0.95
Intracellular DOPAL (*x*_24_)	<0.001	0.95
Intracellular DOPAC (*x*_25_)	<0.001	0.95
DOPAC quinone (*x*_26_)	<0.001	0.95
Superoxide O_2_^-.^ (*x*_27_)	<0.001	0.95
Intracellular hydrogen peroxide H_2_O_2_ (*x*_28_)	<0.001	0.95
hydroxyl radical HO· (*x*_30_)	<0.001	0.95
Peroxynitrite HO·-NO_2_^.^ (*x*_31_)	0.001	0.95
Oxidized glutathione (*x*_33_)	<0.001	0.95
Dehydroascorbate (*x*_34_)	<0.001	0.95

ROC: Receiver operating characteristic, AUC: area under ROC curve

### Biomarkers detected by SVM classifier

Single biomarker obtained through cluster analysis and AUC evaluation can be used for classification (Table 1). However, they can merely be applied to classify the set of etiologies into two groups (Fig 5), and cannot fulfill the requirement of precision medicine. According to the identification of pathogenesis, the disease states of presynaptic dopamine overactivity were caused mainly by the defects of MAO, DAT, and VMAT2. Considering the one-hit and two-hit defects, we applied the SVM classifier to distinguish the disease states at the same dopamine overexpressed level into three classes. Classes 1, 2, and 3 denoted as the defective pairs of (DAT, VMAT2), (DAT, MAO), and (VMAT2, MAO), respectively. Given a dopamine disease level (e.g. 1200) in Fig 4B, 4C and 4D, we uniformly generated 100 samples for each defective pair. According to the ANOVA test, no metabolites were statistically and significantly different among these three classes. To classify these three classes, we employed a machine-learning method with SVM and feature selection proposed in our previous work [40]. In the case study, the metabolic network comprised 34 metabolites. However, all variables were applied to train a SVM classifier that is impractical in clinical trial. Feature selection was used to determine the minimum number of biomarkers. We randomly used 50 samples for training and the other samples for testing. The accuracy of the 50 test samples utilizing SVM with selected biomarkers was first evaluated. The training of SVM and the selection of biomarkers were repeated until the desired accuracy was achieved, and then the optimal biomarkers were obtained. Table 2 shows the optimal biomarkers for a dopamine disease level of 1200 (other dopamine disease levels see S3 Table). The optimal accuracy of classification was 97.7% if three biomarkers were utilized for the features of SVM. By contrast, the optimal accuracy increased to 98.23% if four biomarkers were employed. We could use the optimal biomarkers to accurately assign the patients with presynaptic dopamine overactivity into three classes for furthering the second-line common drug target discovery (Fig 1).

**Table 2:** Selected biomarker and accuracies of the machine-learning method. Dopamine disease level is 1200. 50 samples were randomly selected for training and the rest 50 samples were used for testing. After 100 computations, the average accuracy was listed in this table.

Biomarkers				Accuracy (%)			
				Class 1	Class 2	Class 3	Overall
*x*_8_	*x*_14_	*x*_30_		90.46	99.02	99.96	96.48
*x*_8_	*x*_26_	*x*_34_		92.36	99.96	98.16	96.83
*x*_8_	*x*_21_	*x*_32_		97.64	99.96	95.50	97.70
*x*_6_	*x*_8_	*x*_11_	*x*_21_	96.78	99.00	98.16	97.98
*x*_8_	*x*_13_	*x*_15_	*x*_32_	96.90	99.98	97.82	98.23

*x*_6_: Pyrrolo-quinoline quinine, *x*_8_: Dopamine packed in vesicles, *x*_11_: Extracellular DOPAL, *x*_13_: HVA, *x*_14_: S-Adenosyl-L-homocysteine, *x*_15_: Prostaglandin H_2,_ *x*_21_: Indole-5,6-quinone, *x*_26_: DOPAC quinine, *x*_30_: hydroxyl radical HO·, *x*_32_: Nitrogen dioxide NO_2_, *x*_34_: Dehydroascorbate.

### Discovery of drug targets for different etiologies

In our previous studies [24, 25], we have introduced a fuzzy optimization method to discover the enzyme target in order to recover a disease state caused by only one enzyme defect. The method combined optimization search and decision making and considered therapeutic effects, adverse effects, and target variation effects simultaneously. This study extended this method to discover the first-line common drugs, second-line common drugs, and specific drugs according to different etiologies. The discovery of the first-line common drug targets occurred when the dopamine disease level and a set of etiologies was determined (Fig 1). We considered 33 etiological cases, which consisted of three one-hit defects and uniformly covered all possible combinations of two-hit defects. The fuzzy optimization method was applied to find enzyme targets among 33 etiological cases to achieve the defined objectives. In this case study, the identified enzyme targets are shown in Table 3 for remedying different dopamine disease levels. For the severest disease (*x*_9_ = 1200), we found that a 0.79-fold change (FC) of tyrosine uptake (*u*_*t*_) and 0.32 FC of tyrosine hydroxylase activity (α_1_) were downregulated, and 1.71 FC of COMT activity (α_26_) and 2.74 FC of dehydroascorbate transporter (α_60_) were upregulated to yield 52.03% satisfaction levels for the therapeutic, adverse, and target variation effects. The satisfaction grades obtained from the optimization problem meant that the optimal enzyme targets could remedy the 33 etiological cases, and the worst case among these defects achieved an overall satisfaction grade of 0.52. The satisfaction grades were defined according to a fuzzy membership function (S2 File). This table shows that the lower the overexpression of dopamine disease levels was, the higher the overall satisfaction grade was and the fewer enzyme targets there were. We also considered only the therapeutic effect objective for identifying the first-line common drug targets of each deficiency level; the optimal results are shown in Table 3. Even though the therapeutic objective could achieve a 100% satisfaction grade for all situations, the adverse effect was completely unsatisfactory.

**Table 3:** First-line common drug targets for the set of etiologies.

	Triple objectives				Single objective			
Disease level	T*	A*	V*	Detected target	T*	A	V	Detected target
600	1	0.91	0.91	α_0_, α_8_	1	0	0.35	α_54_
700	1	0.79	0.79	α_0_, α_8_	1	0	0.02	α_23_, α_51_
800	1	0.77	0.77	α_0_, α_8_, α_61_	1	0	0.4	α_0_, α_54_
900	0.97	0.65	0.65	α_0_, α_1_, α_61_	1	0	0.29	α_0_, α_54_
1000	0.88	0.5	0.5	α_0_, α_1_, α_60_	1	0	0.33	α_0_, α_1_, α_54_
1100	0.87	0.63	0.63	α_0_, α_1_, α_8_, α_60_	1	0	0.35	α_0_, α_8_, α_22_, α_50_
1200	0.52	0.52	0.52	α_0_, α_1_, α_26_, α_60_	1	0	0.29	α_0_, α_8_, α_22_, α_42_

T, A, and V denote the satisfaction grade for therapeutic, adverse, and variation effectives of drug target discovery problems, respectively.

The symbol * denotes the optimal grade. The value is obtained from the worst grade among 33 etiological cases. α_j_ is the rate constant of the reaction rate j.

Utilizing the machine-learning classifier with three or four biomarkers, the disease states of each dopamine disease level could be classified into Classes 1, 2, and 3, respectively. For each class of disease state, we considered 12 etiological cases simulated by different dysregulated values to cover all possible combinations of the corresponding two-hit enzyme defects. Here, the drug target discovery problem for each disease case employed the candidate target set Ω^TE^ ∪ Ω^EX^ = {α_0_, α_1_, α_13_, α_14_, α_15_, α_26_, α_60,_ α_61_} obtained from the union of targets in Table 3 in order to find optimal enzyme targets. Consequently, the first-line common drug targets could also be utilized in second-line and specific drugs to avoid the cost of new drug developments. For each class, we discovered three optimal enzyme targets for remedying a dopamine disease level of 1200, and the overall satisfaction grades of Classes 1, 2, and 3 were 0.77, 0.65, and 0.62, respectively (Table 4). Finally, through enzyme kinetic analysis (Fig 1), we could diagnose which enzyme defect was involved, either MAO, DAT, or VMAT2. We then discovered the specific drug targets for each corresponding defect because of specified one-hit or two-hit defects, and obtained the two or three optimal enzyme targets for remedying dopamine 1200 for each specified defect (Table 4, other dopamine disease levels and candidate target set consisted of all enzymes see S4 Table). By considering the therapeutic effect only, we could achieve a 100% satisfaction grade for all situations, but the adverse effect was completely unsatisfactory.

**Table 4:** Second-line common drug targets for class 1, 2 and 3, and the specific drug targets at dopamine disease level of 1200. In the second-line situations, the value is obtained from the worst grade among 12 etiological cases. The drug target discovery problem for each disease case employs the candidate target set Ω^TE^ ∪ Ω^EX^ = {α_0_, α_1_, α_13_, α_14_, α_15_, α_26_, α_60,_ α_61_} obtained from the union of targets in Table 3.

Disease case	Triple objectives				Single objective			
	T*	A*	V*	Detected target	T*	A	V	Detected target
Class 1	0.84	0.77	0.77	α_8_, α_15_, α_26_	0.89	0.51	0.71	α_1_, α_15_
Class 2	0.65	0.65	0.65	α_0_, α_1_, α_14_	0.95	0	0.52	α_1_, α_14_
Class 3	0.95	0.62	0.62	α_0_, α_1_, α_13_	1	0	0.45	α_0_, α_13_
α_13_ (0.64), α_14_ (2.65)	1	0.74	0.74	α_8_, α_15_	1	0	0.7	α_15_, α_60_
α_13_ (0.35), α_15_ (0.73)	1	0.74	0.74	α_0_, α_14_	1	0	0.5	α_14_
α_14_ (2.38), α_15_ (0.73)	1	0.77	0.77	α_0_, α_8_, α_13_	1	0	0.35	α_13_
α_13_ (0.17)	1	0.81	0.81	α_14_, α_15_	1	0.97	0.54	α_14_
α_14_ (3.95)	1	0.75	0.75	α_13_, α_15_	1	0.97	0.33	α_13_
α_15_ (0.22)	1	0.86	0.86	α_0_, α_13_, α_14_	1	0	0.42	α_8_, α_14_

T, A, and V denote the satisfaction grade for therapeutic, adverse, and variation effectives of drug target discovery problems, respectively.

The superscript * denotes as the optimal grade. α_j_ is the rate constant of the reaction rate j. α_i_ (FC) denotes the fold change of enzyme activity between the disease state and the health state.

## Discussion

Schizophrenia is a complex disease with multiple risk factors and causes. Several hypotheses were proposed to explain the etiology of schizophrenia [17]. Presynaptic dopamine overactivity is one of the possible etiologies that have been inferred from the drugs for schizophrenia [46]. However, the causes of presynaptic dopamine overactivity remain under investigation. The proposed pathogenesis detection has identified four one-hit defective enzymes, namely MAO, DAT, VMAT2, and COMT that result in dopamine overexpression. In our simulation, the defect of COMT is almost inactive (−93%) to cause a dopamine level of 600. The involvement of deletions affecting the loci of the COMT gene has been revealed in several studies [47–50]. However, we could not find a feasible result for COMT defect when the dopamine level was greater than 600. Qi et al. [42, 43, 51–53] also revealed that MAO, DAT, VMAT2, and COMT were key components leading to dopamine dysfunction by using extensive Monte Carlo simulations. Nevertheless, our proposed method can identify the precise severity and combinations of these dysregulated enzymes.

The two-hit hypothesis has been applied to hypothesize that cancer is the result of accumulated genetic mutations to a cell [54]. In schizophrenia, the two-hit hypothesis suggests that a combination of genetic susceptibility coupled with a distinct developmental insult can lead to the onset of the full clinical syndrome [45, 55]. Qi el al. [43] reported 50% dopamine excess caused by 9 scenarios of single, double and triple enzyme defects. According to our simulated results of the two-hit defects, we observed that different dysregulated pairs of enzymes caused an overexpression of various dopamine levels (Fig 4). Smaller defects of double enzymes comparing with single enzyme may still cause diseases because of the synergic effect of multiple enzyme dysfunctions. This phenomenon might explain the two-hit hypothesis of schizophrenia.

The major goals of personalized medicine are to predict an individual's susceptibility to developing an illness, achieve an accurate diagnosis, and optimize the most efficient response to treatment [4, 56]. Clinically, schizophrenia is commonly diagnosed on the basis of behavioral signs and symptoms; by contrast, biomarkers have recently been introduced into clinical psychiatry [57, 58]. Biomarkers can aid in staging and classification of the extent of a disease [59] allowing for disease stratification, which is the core concept of precision medicine [17]. In this work, the hierarchical clustering method and AUC could use a single biomarker to roughly classify the disease states into two groups, one with a MAO defect and the other without a MAO defect (Table 1). Furthermore, the SVM classifier was introduced to use three or four biomarkers to accurately categorize the patients with presynaptic dopamine overactivity into three classes to further second-line common drug target discovery (Table 2). The accuracy of classification exceeded 97%. Finally, such information can be provided for an enzyme kinetic analysis to detect which of the specified enzymes cause illness in order to discover a specific drug target.

There are limitations for the application of precision medicine in clinical practice. The traditional medicinal approach relies on a “one-size-fits-all” strategy, or stratified medicine that warrants tailored medicinal care only to a group or subgroup of patients with a known disease. One of the goals of precision medicine is to maximize the probability of curing a disease or ailment while minimizing the potential adverse effects of medicinal interventions in individual patients [60, 61]. In this study, the multiobjective fuzzy optimization formulation was introduced to discover the first-line common drugs, second-line common drugs, and specific drugs that have different etiologies. The approach combined an optimization search and decision making concurrently and simultaneously considered therapeutic effects, adverse effects, and target variation effects to achieve the goals of precision medicine. In the simulated results, we compared the target discovery problem considering with or without adverse effects, and observed that the therapeutic effect could achieve a 100% satisfaction grade if the problem was considered the therapeutic objective only, but the adverse effect was completely unsatisfactory. The multiobjective optimization approach was performed with a trade-off procedure for obtaining a compromise discovery to yield reduced therapeutic effect to improve adverse and target variation effects (Tables 3 and 4).

To identify the second-line and specific drug targets, the candidate target set from the first-line common drug targets was employed. Therefore, we could select different combinations and dosages of remedies among the same drugs targets, rather than develop new medications for common and specific drug targets. The satisfaction grades of first-line common drug targets are lower than that of second-line and specific drug targets. However, medications of first-line common drug targets can treat disease states with different etiologies when biomarkers are not available. As more biomarkers and enzyme activities can be examined, higher satisfaction grades can be obtained by second-line and specific drug targets (Table 4).

The SOAP note is a problem oriented medical record (Fig 1). Optimization processes in SOAP, i.e. pathogenesis identification, biomarker detection, and drug discovery, should be conducted in advance by biomedical scientists. When treating a patient, a physician just need to measure dopamine level, check biomarkers, and then choose the optimal drugs according to the protocol. Further tests, such as DNA sequencing, gene expression levels and metabolic levels might be helpful to define pathological states for specific drugs. However, physicians can still use common drugs if advanced tests are not available. The disease states may be altered by remedy, and therefore it is indicated to follow up these tests periodically.

In our previous studies [24, 25], we introduced a fuzzy multiobjective optimization method for drug discovery. In this study, we extended this method to pathogenesis identification, biomarker detection, and designs of common and specific drugs. In addition, we implemented these results to draw a SOAP flowchart that can be employed by physicians in clinical practice easily. However, we would like to emphasize that the accuracy of this method depends on the accuracy of the model. For example, the model we used in this study focus on the presynaptic dopamine metabolism, but schizophrenia is also related to glutamatergic, GABAergic, glycinergic and serotonergic systems [62]. If these complex systems could be enrolled in the model, the proposed optimization approaches can be applied to the whole process of precision medicine.

## Conclusions

This study proposed a strategy of model-based optimization for precision medicine to remedying presynaptic dopamine overexpression. The strategy consisted of pathogenesis detection, biomarker identification, enzyme kinetic analysis, and drug target discovery for treating a patient. The procedures were similar to SOAP notes used by physicians and other healthcare professionals. One-hit and two-hit enzyme defects were detected by the pathogenesis problem to determine a set of etiologies, which was similar to the results obtained by Monte Carlo computations [51]. The identified biomarkers were then applied to classify such etiologies into three classes. Moreover, the specific enzyme defects could be identified if experiments for enzyme kinetic analysis were available. Finally, the multiobjective fuzzy optimization formulation was introduced to discover the first-line common drugs, second-line common drugs, and specific drugs for different etiologies. The approach combined optimization search and decision making concurrently and considered therapeutic effects, adverse effects, and target variation effects simultaneously to achieve the goals of precision medicine.

## Supporting information

S1 Table Relative sensitivity. The relative sensitivity of dopamine concentration with respect to each enzyme activity.
(XLSX)

S2 Table Single biomarker. Candidates of biomarkers selected by cluster analysis and ROC curves for different dopamine disease levels.
(XLSX)

S3 Table Multiple biomarkers. Selected biomarkers and accuracies obtained by the machine-learning method for different dopamine disease levels.
(XLSX)

S4 Table Optimal enzyme targets. Drug target discovery problem for second-line common drug targets and the specific drug targets using candidate target set consisted of all enzymes.
(XLSX)

S1 File NHDE algorithm. The detailed computational procedure of NHDE.
(DOCX)

S2 File Solving strategy. Definition of the symbols in the pathogenesis problem and fuzzy multiobjective target discovery problem and its solving strategy.
(DOCX)

S3 File Presynaptic dopamine model. List of dependent and independent variables and kinetic equations for presynaptic dopamine metabolic model.
(DOCX)

S4 File Glossary of clinical terms. List of definitions of clinical terms.
(DOCX)
