# Supplementary material for: Model-based optimization approaches for precision medicine: A case study in presynaptic dopamine overactivity
Source: PLoS One. 2017 Jun 14;12(6):e0179575. doi: 10.1371/journal.pone.0179575 (PMC5470743; doi:10.1371/journal.pone.0179575)
Supplement: S2 File — Definition of the symbols in the pathogenesis problem and fuzzy multiobjective target discovery problem and its solving strategy. (DOCX) [file pone.0179575.s006.docx]

Model-based optimization approaches for precision medicine: A case study in presynaptic dopamine overactivity

Kai-Cheng Hsu^1^ and Feng-Sheng Wang^2^*

^1^Department of Neurology, National Taiwan University Hospital Yunlin Branch, Yunlin 64041, Taiwan

^2^Department of Chemical Engineering, National Chung Cheng University, Chiayi 62102, Taiwan

Email: Kai-Cheng Hsu - [edwardfirst@gmail.com](mailto:edwardfirst@gmail.com); Feng-Sheng Wang* - [chmfsw@ccu.edu.tw](mailto:chmfsw@ccu.edu.tw)

**Supplementary file 2:**

Definition of the symbols in the pathogenesis problem and fuzzy multiobjective target discovery problem and its solving strategy.

Pathogenesis problem:

$\begin{matrix} \min_{\mathbf{x,}\hat{\mathbf{\alpha}}\mathbf{,u},\mathbf{z}} \sum_{i\in\Omega^{DS}} \left(x_{i}-x_{i}^{disease}\right)^{2}+\sum_{j\in\Omega^{Enz}} \left(1-\hat{\alpha}_{j}/\hat{\alpha}_{j}^{basal}\right)^{2}+\sum_{j\in\Omega^{EX}} \left(1-u_{j}/u_{j}^{basal}\right)^{2} \\ \text{subjec to} \\ \text{Material balance equations:} \\ \left\{\begin{matrix} \sum_{j=1}^{r} N_{ij}v_{j}\left(\mathbf{x,\alpha}\right)+\sum_{j=1}^{m} B_{ij}u_{j}=0,i\in\Omega^{SP} \\ w_{j}=\hat{\alpha}_{j}+\sum_{k=1}^{n_{v}} g_{jk}y_{k},j\in\Omega^{Rxn} \\ v_{j}=\exp\left(w_{j}\right)\text{ } \\ x_{i}=\exp\left(y_{i}\right)\text{ } \\ \text{Metabolite constraints:} \\ x_{i}^{LB}\leq x_{i}\leq x_{i}^{UB}; i\in\Omega^{SP} \end{matrix}\right. \\ \\ \left\{\begin{matrix} \text{Target constraints: } \\ \text{up-regulation: }\left\{\begin{matrix} \hat{\alpha}_{z}^{basal}\leq\hat{\alpha}_{z}\leq\hat{\alpha}_{z}^{UB},z\in\Omega_{z}^{up} \\ u_{z}^{basal}\leq u_{z}\leq u_{z}^{UB}\text{ } \end{matrix}\right. \\ \text{down-regulation: }\left\{\begin{matrix} \hat{\alpha}_{z}^{LB}\leq\hat{\alpha}_{z}\leq\hat{\alpha}_{z}^{basal},z\in\Omega_{z}^{down} \\ u_{z}^{LB}\leq u_{z}\leq u_{z}^{UB} \end{matrix}\right.\text{ } \\ \hat{\alpha}_{z}=\hat{\alpha}_{z}^{basal},z\notin\Omega_{z}=\Omega_{z}^{up}+\Omega_{z}^{down} \end{matrix}\right. \end{matrix}$ (S1)

Fuzzy multiobjective target discovery (FMTD) problem:

$\left\{\begin{matrix} \text{Therapeutic effect:}\mathop{Fuzzy equal}\limits_{\mathbf{x,}\hat{\mathbf{\alpha}}\mathbf{,u},\mathbf{z}} x_{id}\approx x_{i}^{basal}; i\in\Omega^{TH},d\in\Omega^{DS} \\ \text{Adverse effect:}\mathop{Fuzzy \min}\limits_{\mathbf{x,}\hat{\mathbf{\alpha}}\mathbf{,u},\mathbf{z}} x_{jd}; j\in\Omega^{AE},d\in\Omega^{DS} \\ \text{Variation effect:}\mathop{Fuzzy equal}\limits_{\mathbf{x},\hat{\mathbf{\alpha}}\mathbf{,u},\mathbf{z}} \hat{\alpha}_{z_{k}}\approx\hat{\alpha}_{z_{k}}^{basal}; z_{k}\in\Omega^{TE}=\Omega^{Rxn}\backslash\Omega^{DS} \\ \text{ }\mathop{Fuzzy equal}\limits_{\mathbf{x,}\hat{\mathbf{\alpha}}\mathbf{,u},\mathbf{z}} u_{z_{k}}\approx u_{z_{k}}^{basal}; z_{k}\in\Omega^{EX} \\ \text{Number of targets: }\min_{\mathbf{x,}\hat{\mathbf{\alpha}}\mathbf{,u},\mathbf{z}} \sum_{k} z_{k};\text{ }z_{k}\in\Omega^{TE} \\ \\ \left\{\begin{matrix} \text{Material balance equations:} \\ \sum_{j=1}^{r} N_{ij}v_{jd}\left(\mathbf{x},\mathbf{\alpha}\right)+\sum_{j=1}^{m} B_{ij}u_{j}=0; i\in\Omega^{SP},d\in\Omega^{DS} \\ \text{Metabolite constraints:} \\ x_{i}^{LB}\leq x_{id}\leq x_{i}^{UB} \end{matrix}\right. \\ \left\{\begin{matrix} \text{Target constraints:} \\ \hat{\alpha}_{z_{k}}^{LB}\leq\hat{\alpha}_{z_{k}}\leq\hat{\alpha}_{z_{k}}^{UB}; z_{k}\in\Omega^{TE} \\ \hat{\alpha}_{z_{k}}=\hat{\alpha}_{z_{k}}^{basal}; z_{k}\notin\Omega^{TE} \\ u_{z_{k}}^{LB}\leq u_{z_{k}}\leq u_{z_{k}}^{UB};z_{k}\in\Omega^{EX} \\ u_{z_{k}}=u_{z_{k}}^{basal};z_{k}\notin\Omega^{EX} \end{matrix}\right. \end{matrix}\right.$ (S2)

The *i*^th^ reaction rate, *v_id_*, depends on each disease state and is expressed in the power law function as following

$v_{jd}=\alpha_{jd}\prod_{k=1}^{n} x_{k}^{g_{jk}}$ (S3)

$$\alpha_{jd}=\left\{\begin{matrix} \alpha_{j}, j\in\Omega^{TE}\text{ and }j\notin\Omega^{DS} \\ \alpha_{j}^{basal}, j\in\Omega^{DS}\text{ and }j\neq d \\ \alpha_{j}^{DS}, j\in\Omega^{DS}\text{ and }j=d \end{matrix}\right.$$

Notation:

| Symbol | Definition |
| --- | --- |
| *B_ij_* | The connectivity matrix describing the corresponding control *ij* |
| *N_ij_* | The stoichiometric matrix describing the interconnecting fluxes *ij* |
| *g_jk_* | The kinetic order *jk* |
| *u_j_* | The external control *j* |
| *u_j_^basal^* | The external control *j* at the basal (healthy) level |
| $u_{z}$ | The external control *z* |
| $u_{z}^{basal}$ | The external control *z* at the basal (healthy) level |
| $u_{z}^{LB}$ | The external control *z* at the lower bound |
| $u_{z}^{UB}$ | The external control *z* at the upper bound |
| $u_{z_{k}}$ | The external control *z_k_* |
| $u_{z_{k}}^{basal}$ | The external control *z_k_* at the basal (healthy) level |
| $u_{z_{k}}^{LB}$ | The external control *z_k_* at the lower bound |
| $u_{z_{k}}^{UB}$ | The external control *z_k_* at the upper bound |
| *v_j_* | The rate constant of the reaction rate *j* |
| *w_j_* | The logarithmic reaction rate *j* |
| *x_i_* | The concentration of the metabolite *i* in the metabolic network |
| $x_{i}^{basal}$ | The concentration of the metabolite *i* at the basal (healthy) level |
| *x_i_^disease^* | The concentration of the metabolite *i* at the disease state in the metabolic network |
| $x_{i}^{LB}$ | The concentration of the metabolite *i* at the lower bound |
| $x_{i}^{UB}$ | The concentration of the metabolite *i* at the upper bound |
| $x_{id}$ | The concentration of the metabolite *i* in the disease state |
| $x_{jd}$ | The concentration of the metabolite *j* in the disease state |
| *y_k_* | The logarithmic concentration *k* |
| $z_{k}$ | The number of targets |
| $\hat{\alpha}_{j}$ | The logarithmic rate constant of the reaction rate *j* |
| $\hat{\alpha}_{j}^{basal}$ | The logarithmic rate constant of the reaction rate *j* at the basal (healthy) level |
| $\hat{\alpha}_{z_{k}}^{LB}$ | The logarithmic rate constant of the reaction rate *z_k_* at the lower bound |
| $\hat{\alpha}_{z_{k}}^{UB}$ | The logarithmic rate constant of the reaction rate *z_k_* at the upper bound |
| $\hat{\alpha}_{z}$ | The logarithmic rate constant of the reaction rate *z* |
| $\hat{\alpha}_{z}^{basal}$ | The logarithmic rate constant of the reaction rate *z* at the basal (healthy) level |
| $\hat{\alpha}_{z}^{LB}$ | The logarithmic rate constant of the reaction rate *z* at the lower bound |
| $\hat{\alpha}_{z}^{UB}$ | The logarithmic rate constant of the reaction rate *z* at the upper bound |
| $\hat{\alpha}_{z_{k}}$ | The logarithmic rate constant of the reaction rate *z_k_* |
| $\hat{\alpha}_{z_{k}}^{basal}$ | The logarithmic rate constant of the reaction rate *z_k_* at the basal (healthy) level |
| Ω*^DS^* | The set of disease metabolites |
| Ω*^Enz^* | The set of enzyme targets |
| Ω*^EX^* | The set of external controls |
| Ω*^SP^* | The set of species |
| Ω*^Rxn^* | The set of reactions |
| $\Omega_{z}^{up}$ | The set of up-regulation reactions |
| $\Omega_{z}^{down}$ | The set of down-regulation reactions |
| $\Omega^{TH}$ | The set of fuzzy equal objectives for therapeutic effects |
| $\Omega^{AE}$ | The set of fuzzy minimization objectives for adverse effects |
| $\Omega^{TE}$ | The set of manipulated variables |
| $\Omega^{Rxn}$ | The set of n_v_ -dimentional reaction rates |
| $\Omega^{EX}$ | The set of n_u_ -dimentional external control variable |

**Strategy for solving fuzzy multiobjective target discovery problem**

To solve the FMTD framework (S1), we defined a membership function for each fuzzy equal objective and fuzzy minimizing objective for quantifying each corresponding satisfaction grade. The generalized membership function for each fuzzy equal objective is described in Eq. (S4) as follows:

$\eta_{i}(x_{i})\text{ }=\left\{\begin{matrix} 0, x_{i}\leq x_{i}^{LB} \\ d_{i}^{xL}, x_{i}^{LB}\leq x_{i}\leq x_{i}^{basal,LB} \\ 1, x_{i}^{basal,LB}\leq x_{i}\leq x_{i}^{basal,UB} \\ d_{i}^{xR}, x_{i}^{basal,UB}\leq x_{i}\leq x_{i}^{UB} \\ 0, x_{i}\geq x_{i}^{UB} \end{matrix}\right.$ (S4)

The left-hand side membership function is a strictly monotonically increasing function, $d_{i}^{xL}$, whereas the right-hand side is a strictly monotonically decreasing function, $d_{i}^{xR}$. A membership function is similar to assess the effects of inaccuracies in external control variables and reaction rate constants. Monte Carlo simulation is generally applied to assess such an experimental imprecision. However, a designer can define a membership function for fuzzy optimization problems in advance. Sakawa [1] proposed five types of membership functions, namely linear, exponential, hyperbolic, inverse, and piecewise linear functions, for quantifying the behavior of fuzzy objectives or the constraints. Here, $x_{i}^{LB}$ and $x_{i}^{UB}$ are the lower and upper bounds of the *i*^th^ metabolite concentration or enzyme activity provided by the designer. The satisfaction grade is zero when the metabolite concentration or enzyme activity is beyond its lower or upper bounds. The satisfaction grade or membership function value is equal to one when the corresponding metabolite concentration or enzyme activity is between the lower and upper bounds of the basal value, represented as $x_{i}^{basal,LB}$, $x_{i}^{basal,UB}$, and $x_{i}^{basal}$, respectively. The satisfaction grade is between zero and one when the metabolite concentration or enzyme activity is within its range on the left- or right-hand side membership function. For each fuzzy minimizing objective, the membership function is defined as a strictly monotonically decreasing function on the right-hand side.

$\eta_{j}(x_{j})\text{ }=\left\{\begin{matrix} 1, x_{j}\leq x_{i}^{basal} \\ d_{i}^{xR}, x_{i}^{basal}\leq x_{i}\leq x_{i}^{UB} \\ 0, x_{i}\geq x_{i}^{UB} \end{matrix}\right.$ (S5)

According to the membership functions expressed in Eqs. (S4) and (S5), we conclude that the intersection for these membership functions is zero when either the fuzzy equal objective functions are outside the corresponding lower and upper bounds or the fuzzy minimizing objectives are greater than the corresponding upper bounds. By contrast, when all objectives are within their corresponding bounds, the intersection for all membership functions should show a certain degree of satisfaction. For each membership function being introduced by the designer, the FMTD problem is designed for determining a maximum intersection for all membership functions between the desired bounds. The FMTD framework is then transferred to the maximizing decision framework, which is a discontinuous function. The detailed procedures have been discussed previously [2, 3]. Thus, the maximizing decision problem can be rewritten as an equivalent optimization problem on the solving domain to avoid a discontinuous computation, as follows:

$\begin{matrix} \max_{\lambda,(\mathbf{x},\hat{\mathbf{\alpha}},\mathbf{u},\mathbf{z})\in\Psi} \left(\frac{Z^{UB}-\sum z_{j}}{Z^{UB}-1}\right)\lambda\\ \eta_{i}\left(x_{i}\right)\geq\lambda, i\in\Omega^{TH},d\in\Omega^{DS} \\ \eta_{j}\left(x_{j}\right)\geq\lambda, j\in\Omega^{AE} \\ \eta_{k}\left(\hat{\alpha}_{k}\right)\geq\lambda, k\in\Omega^{TE} \\ \eta_{l}\left(u_{l}\right)\geq\lambda,l\in\Omega^{EX} \end{matrix}$ (S6)

where the crisp feasible domain Ψ includes the kinetic model and inequality constraints described in Eqs. (S2) and (S3), respectively. The total number of the identified enzyme targets is the crisp objective so that it is transformed to a normalized value as expressed in Eq. (S6), where Z^UB^ is the upper bound. The advantage of this method is that the optimal membership grade corresponds to the satisfaction level for each objective, and the optimal decision λ represents the overall satisfaction grade (equivalent to the lower bound) of the problem. The maximizing decision problem can be then solved by the NHDE algorithm.

**References**

1. Sakawa M: **Fuzzy sets and interactive multiobjective optimization**. New York: Plenum; 1993. pp. 308.
2. Hsu KC, Wang FS: **Fuzzy optimization for detecting enzyme targets of human uric acid metabolism.** Bioinformatics 2013, 29, 3191-3198.
3. Hsu KC, Wang FS: **Fuzzy decision making approach to identify optimum enzyme targets and drug dosage for remedying presynaptic dopamine deficiency**. PLOS ONE, 2016, Oct 13, 1-18
