# Supplementary material for: Model-based optimization approaches for precision medicine: A case study in presynaptic dopamine overactivity
Source: PLoS One. 2017 Jun 14;12(6):e0179575. doi: 10.1371/journal.pone.0179575 (PMC5470743; doi:10.1371/journal.pone.0179575)
Supplement: S3 File — List of dependent and independent variables and kinetic equations for presynaptic dopamine metabolic model. (DOCX) [file pone.0179575.s007.docx]

Model-Based Optimization Approaches for Precision Medicine: A Case Study in Presynaptic Dopamine Overactivity

Kai-Cheng Hsu^1^ and Feng-Sheng Wang^2^*

^1^Department of Neurology, National Taiwan University Hospital Yunlin Branch, Yunlin 64041, Taiwan

^2^Department of Chemical Engineering, National Chung Cheng University, Chiayi 62102, Taiwan

Email: Kai-Cheng Hsu - [edwardfirst@gmail.com](mailto:edwardfirst@gmail.com); Feng-Sheng Wang* - [chmfsw@ccu.edu.tw](mailto:chmfsw@ccu.edu.tw)

**Supplementary file 3:**

The kinetic model of the presynaptic dopamine metabolic pathway (Figure S3-1) was modified from Qi *et al*. (2008). Detailed lists of all metabolites, variable names in the model, and abbreviations are presented in Supplementary Table S3-1. Metabolites for the therapeutic effect are highlighted in red, whereas toxic species, reactive oxygen and nitrogen species for adverse effects are highlighted in yellow.


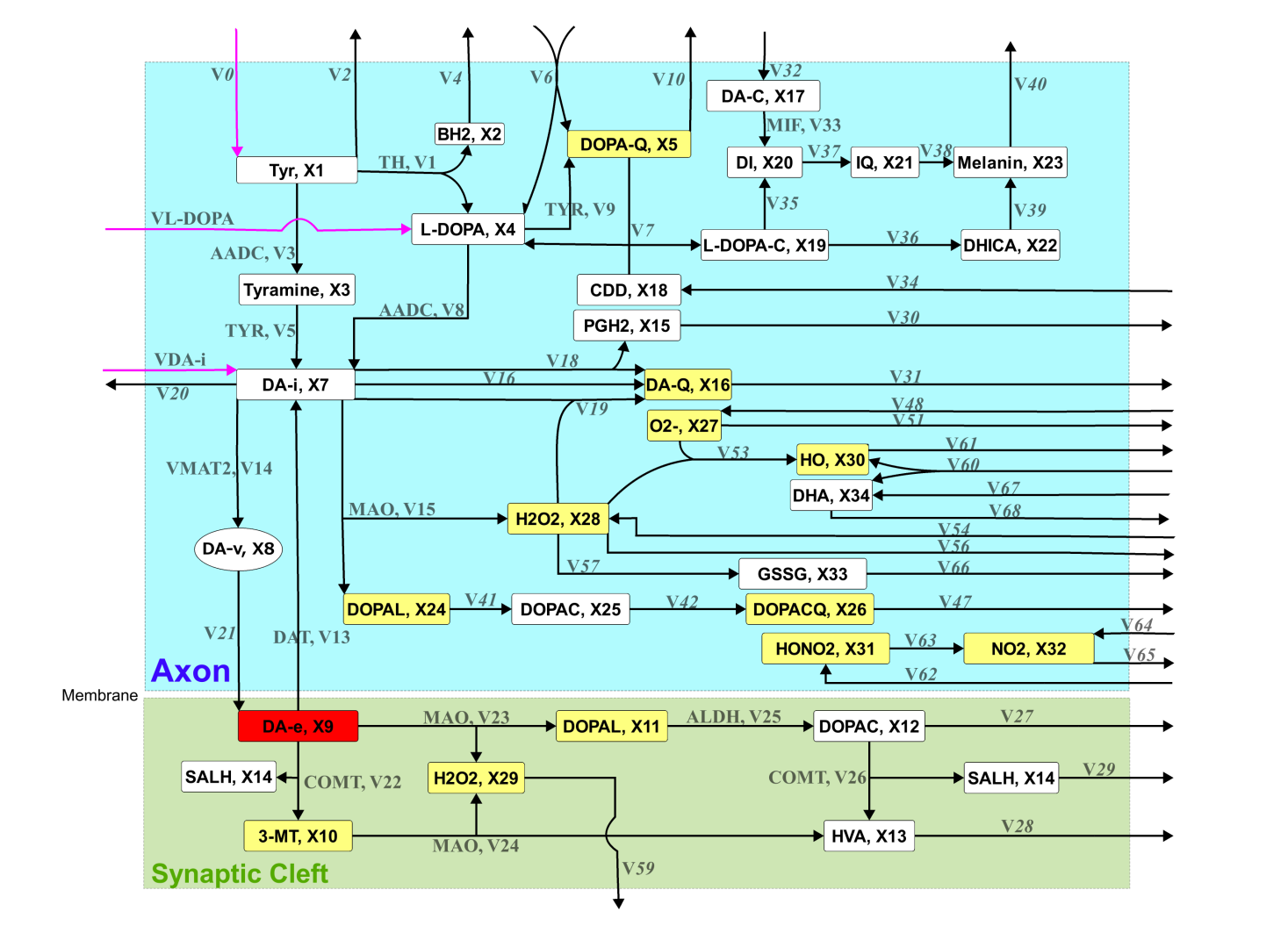
**Figure S3-1.** Schematic network diagram of the presynaptic dopamine metabolic pathway.

**Table S3-1**. ID and name of dependent and independent metabolites.

| Variable (ID) | Name |
| --- | --- |
| Time dependent metabolites | |
| x1 | Tyrosine |
| x2 | Dihydrobiopterin |
| x3 | Tyramine |
| x4 | L-DOPA produced from tyrosine |
| x5 | Dopaquinone |
| x6 | Pyrrolo-quinoline quinone |
| x7 | Intracellular dopamine |
| x8 | Dopamine packed in vesicles |
| x9 | Extracellular dopamine |
| x10 | 3-Methoxytyramine |
| x11 | Extracellular DOPAL |
| x12 | Extracellular DOPAC |
| x13 | HVA |
| x14 | S-Adenosyl-L-homocysteine |
| x15 | Prostaglandin H2 |
| x16 | Dopamine quinone |
| x17 | Dopamine chrome |
| x18 | 2-Carboxy-2,3-dihydro-5,6-dihydroxyindole |
| x19 | L-Dopachrome |
| x20 | 5,6-Dihydroxyindole |
| x21 | Indole-5,6-quinone |
| x22 | 5,6-Dihydroxyindole-2-carboxylate |
| x23 | Melanin |
| x24 | Intracellular DOPAL |
| x25 | Intracellular DOPAC |
| x26 | DOPAC quinone |
| x27 | Superoxide O2 − . |
| x28 | Intracellular hydrogen peroxide H2O2 |
| x29 | Extracellular hydrogen peroxide H 2 O 2 |
| x30 | hydroxyl radical HO· |
| x31 | Peroxynitrite HO· — NO2 . |
| x32 | Nitrogen dioxide NO2 |
| x33 | Oxidized glutathione |
| x34 | Dehydroascorbate |
| Non-time dependent metabolites | |
| x35 | Tyrosine hydroxylase |
| x36 | Tyrosinase |
| x37 | Prostaglandin G/H synthase |
| x38 | XO xanthine oxidase |
| x39 | ALDH aldehyde dehydrogenase |
| x40 | Monoamine oxidase |
| x41 | Semicarbazide-sensitive amine oxidase |
| x42 | DOPA decarboxylase |
| x43 | Dopachrome isomerase |
| x44 | Catalase |
| x45 | Superoxide dismutase |
| x46 | Extracellular aldehyde dehydrogenase |
| x47 | Extracellular monoamine oxidase |
| x48 | Catechol O-methyltransferase |
| x49 | Glutathione peroxidase |
| x50 | Glutathione reductase |
| x51 | Extracellular semicarbazide-sensitive amine oxidas |
| x52 | Migration inhibitory factor |

**Table S3-2**. Reaction equation and its corresponding identifier in the metabolic network.

| Identifier | Enzyme | Reaction Equation |
| --- | --- | --- |
| uldopa | L-DOPA input | -> x4 |
| udopamine | dopamine input | -> x7 |
| utyrosine | tyrosine input | -> x1 |
| v1 | tyrosine hydroxylase (TH) | x1 -> x2 + x4 |
| v2 | tyrosine output | x1 -> |
| v3 | DOPA decarboxylase (AADC) | x1 -> x3 |
| v4 | BH2 output | x2 -> |
| v5 | tyrosinase (TYR) | x3 -> x7 |
| v6 |  | -> x4 + x5 |
| v7 |  | x5 + x18 -> x4 + x19 |
| v8 | DOPA decarboxylase (AADC) | x4 -> x7 |
| v9 | tyrosinase (TYR) | x4 -> x5 |
| v10 |  | x5 -> |
| v11 |  | -> x6 |
| v12 |  | x6 -> |
| v13 | dopamine transporter(DAT) | x9 -> x7 |
| v14 | vesicular monoamine transporter(VMAT2) | x7 -> x8 |
| v15 | monoamine oxidase(MAO), semicarbazide-sensitive amine oxidase (SSAO) | x7 -> x24 + x28 |
| v16 | autoxidation, TYR, X53, XO | x7 -> x16 |
| v17 | autoxidation, TYR, X53, XO | x7 -> x16 |
| v18 | autoxidation, TYR, X53, XO | x7 -> x15 + x16 |
| v19 |  | x7 + x28 -> x16 |
| v20 |  | x7 -> |
| v21 | vesicle secretion | x8 -> x9 |
| v22 | catechol-O-methyltransferase(COMT) | x9 -> x10 + x14 |
| v23 | MAO, SSAO | x9 -> x11 + x29 |
| v24 | ALDH, MAO | x10 -> x13 + x29 |
| v25 | aldehyde dehydrogenase (ALDH) | x11 -> x12 |
| v26 | catechol-O-methyltransferase(COMT) | x12 -> x13 + x14 |
| v27 |  | x12 -> |
| v28 |  | x13 -> |
| v29 |  | x14 -> |
| v30 |  | x15 -> |
| v31 |  | x16 -> |
| v32 |  | -> x17 |
| v33 | migration inhibitory factor (MIF) | x17 -> x20 |
| v34 |  | -> x18 |
| v35 | tyrosinase (TYR) | x19 -> x20 |
| v36 | dopachrome isomerase (DCT) | x19 -> x22 |
| v37 | tyrosinase (TYR) | x20 -> x21 |
| v38 |  | x21 -> x23 |
| v39 |  | x22 -> x23 |
| v40 |  | x23 -> |
| v41 | aldehyde dehydrogenase (ALDH) | x24 -> x25 |
| v42 | tyrosinase (TYR) | x25 -> x26 |
| v43 | tyrosinase (TYR) | x25 -> x26 |
| v44 | tyrosinase (TYR) | x25 -> x26 |
| v45 | tyrosinase (TYR) | x25 -> x26 |
| v46 | tyrosinase (TYR) | x25 -> x26 |
| v47 |  | x26 -> |
| v48 |  | -> x27 |
| v49 |  | -> x27 |
| v50 |  | -> x27 |
| v51 |  | x27 -> |
| v52 |  | x27 -> |
| v53 |  | x27 + x28 -> x30 |
| v54 |  | -> x28 |
| v55 |  | -> x28 |
| v56 |  | x28 -> |
| v57 |  | x28 -> x33 |
| v58 |  | x28 -> |
| v59 |  | x29 -> |
| v60 |  | -> x30 + x34 |
| v61 |  | x30 -> |
| v62 |  | -> x31 |
| v63 |  | x31 -> x32 |
| v64 |  | -> x32 |
| v65 |  | x32 -> |
| v66 |  | x33 -> |
| v67 |  | -> x34 |
| v68 |  | x34 -> |

The rate equations were obtained from Qi *et al*. (2008). The value for each rate constant was evaluated at the healthy state.

| Rate equations | Rate law |
| --- | --- |
| uldopa | uldopa |
| udopamine | udopamine |
| utyrosine | utyrosine |
| v1 | 12.34409 (X1^0.5) (X4^-0.3) (X5^-0.3) (X7^-0.3) (X16^-0.3) X35 |
| v2 | 0.0005656854 (X1^0.5) (X4^0.5) X36 |
| v3 | 0.01009356 (X1^0.5) (X7^-0.3) X42 |
| v4 | 300.4164 (X2^0.5) |
| v5 | 0.001825742 (X3^0.5) (X4^0.5) X36 |
| v6 | 0.0002828427 (X1^0.5) (X4^0.5) X36 |
| v7 | 2.767677 (X5^0.5) (X18^0.5) |
| v8 | 6.670782 (X4^0.5) (X7^-0.3) X42 |
| v9 | 0.3478394 X4 (X30^0.5) |
| v10 | 24.75485 (X5^0.5) |
| v11 | 18.2567 (X5^0.5) |
| v12 | 9.128352 (X6^0.5) |
| v13 | 4.198429 X9 |
| v14 | 2.998878 X7 |
| v15 | 0.001785214 (X7^0.5) X40 X41 |
| v16 | 0.03710922 X7 (X30^0.5) |
| v17 | 0.0009917856 (X7^0.5) X36 |
| v18 | 0.0009917856 (X7^0.5) X37 |
| v19 | 0.00044354 (X7^0.5) (X28^0.5) X38 |
| v20 | 0.2975357 (X7^0.5) |
| v21 | 0.03894646 X8 |
| v22 | 0.6772013 (X9^0.5) (X12^-0.3) (X14^-0.3) X48 |
| v23 | 0.001049607 (X9^0.5) X47 X51 |
| v24 | 0.004693986 (X10^0.5) X46 X47 |
| v25 | 2.642754 (X11^0.5) (X12^-0.3) X46 |
| v26 | 1.642312 (X9^-0.3) (X12^0.5) (X14^-0.3) X48 |
| v27 | 4.198429 (X12^0.5) |
| v28 | 30.85202 (X13^0.5) |
| v29 | 168.9835 (X14^0.5) |
| v30 | 1.173497 (X15^0.5) |
| v31 | 14.10638 (X16^0.5) |
| v32 | 12.69575 (X16^0.5) |
| v33 | 0.1269575 (X17^0.5) X52 |
| v34 | 6.188713 (X5^0.5) |
| v35 | 0.005569842 (X4^0.5) (X19^0.5) X36 |
| v36 | 0.006188713 (X19^0.5) X43 |
| v37 | 0.02352434 (X4^0.5) (X20^0.5) X36 |
| v38 | 23.52434 (X21^0.5) |
| v39 | 0.6188713 (X22^0.5) |
| v40 | 17.07183 (X23^0.5) |
| v41 | 15.07592 (X24^0.5) (X25^-0.3) X39 |
| v42 | 0.3578389 X25 (X30^0.5) |
| v43 | 0.02677821 (X25^0.5) X36 |
| v44 | 0.004463035 (X25^0.5) X45 |
| v45 | 0.3155842 (X25^0.5) (X31^0.5) |
| v46 | 0.6311685 (X25^0.5) (X32^0.5) |
| v47 | 149.3617 (X26^0.5) |
| v48 | 0.6956788 X4 (X30^0.5) |
| v49 | 0.07421844 X7 (X30^0.5) |
| v50 | 0.7156778 X25 (X30^0.5) |
| v51 | 3.065113 (X27^0.5) X45 |
| v52 | 38.13551 X27 |
| v53 | 0.171345 X27 X28 |
| v54 | 1.532556 (X27^0.5) X45 |
| v55 | 33.41228 X27 |
| v56 | 1.308324 (X28^0.5) X44 |
| v57 | 1.308324 (X28^0.5) X49 |
| v58 | 78.01336 X28 |
| v59 | 296.8737 (X29^0.5) |
| v60 | 39.00668 X28 |
| v61 | 140.9384 (X30^0.5) |
| v62 | 4.723232 X27 |
| v63 | 0.01686869 X25 X31 |
| v64 | 23.61616 |
| v65 | 0.03373737 X25 X32 |
| v66 | 0.2925501 (X33^0.5) X50 |
| v67 | 16.70614 X27 |
| v68 | 27.85641 (X34^0.5) |

The material balance equations in the metabolic network are expressed as follows:

| x1' = utyrosine –v1 –v2 –v3 |
| --- |
| x2' = v1 –v4 |
| x3' = v3 –v5 |
| x4' = uldopa + v1 + v6 + v7 - v8- v9 |
| x5' = v9 + v6 - v10 - v7 |
| x6' = v11 - v12 |
| x7' = udopamine + v8 + v5 + v13 - v14 - v15 - v16 - v17 - v18 - v19 - v20 |
| x8' = v14 - v21 |
| x9' = v21 - v22 - v23 - v13 |
| x10' = v22 - v24 |
| x11' = v23 - v25 |
| x12' = v25 - v26 - v27 |
| x13' = v24 + v26 -v28 |
| x14' = v22 + v26 - v29 |
| x15' = v18 - v30 |
| x16' = v16 + v17 + v18 + v19 - v31 |
| x17' = v32 - v33 |
| x18' = v34 - v7 |
| x19' = v7 - v35 - v36 |
| x20' = v35 + v33 - v37 |
| x21' = v37 - v38 |
| x22' = v36 - v39 |
| x23' = v38 + v39 - v40 |
| x24' = v15 - v41 |
| x25' = v41 - v42 - v43 - v44 - v45 - v46 |
| x26' = v42 + v43 + v44 + v45 + v46- v47 |
| x27' = v48 + v49 + v50 - v51 -v52 - v53 |
| x28' = v15 + v54 + v55 - v56 - v57 -v58 - v53 - v19 |
| x29' = v24 + v23 - v59 |
| x30' = v60 +v53 - v61 |
| x31' = v62 - v63 |
| x32' = v64 + v63 - v65 |
| x33' = v57 - v66 |
| x34' = v67 + v60 -v68 |

**Table S3-3.** Concentrations (relative unit) of metabolites at healthy state

| Metabolite | HS | Objective |
| --- | --- | --- |
| Extracellular dopamine (x_9_) | 400 | Therapeutic |
| Dopaquinone (x_5_) | 5 | Toxic |
| 3-Methoxytyramine (x_10_) | 20 | Toxic |
| Extracellular DOPAL (x_11_) | 10 | Toxic |
| Dopamine quinone (x_16_) | 10 | Toxic |
| Intracellular DOPAL (x_24_) | 5 | Toxic |
| DOPAC quinone (x_26_) | 10 | Toxic |
| Superoxide O_2_^−^ (x_27_) | 5 | ROS |
| Intracellular hydrogen peroxide H_2_O_2_ (x_28_) | 5 | ROS |
| Extracellular hydrogen peroxide H_2_O_2_ (x_29_) | 2 | ROS |
| hydroxyl radical HO^-^ (x_30_) | 2 | ROS |
| Peroxynitrite NO_3_^-^ (x_31_) | 2 | RNS |
| Nitrogen dioxide NO2 (x_32_) | 2 | RNS |
