# Supplementary material for: Model-based optimization approaches for precision medicine: A case study in presynaptic dopamine overactivity
Source: PLoS One. 2017 Jun 14;12(6):e0179575. doi: 10.1371/journal.pone.0179575 (PMC5470743; doi:10.1371/journal.pone.0179575)
Supplement: S4 File — List of definitions of clinical terms. (DOCX) [file pone.0179575.s008.docx]

**Glossary of Clinical Terms:**

**Precision medicine:** Treatments tailored to individual patients on the basis of their genetic, biomarker, phenotypic, and psychosocial characteristics.

**Therapeutic effect:** The main goal of treatments to heal the disease.

**Adverse effect:** The side effects caused by diseases or treatments.

**Pathogenesis:** The causes of diseases.

**Biomarker:** A measurable indicator of some biological state or condition.

**Drug target:** The key enzyme in the model for drug actions.

**SOAP notes:** A problem oriented medical record including: Subjective (S): The subjective complains of a patient; Objective (O): The objective observations or tests of a patient; Assessment (A): The diagnosis or evaluation of a patient; Plan (P): The decisions made by physicians for a patient, including further exams or treatments.

**Nigrostriatal dopamine network:** The key dysfunctional area in the brain related to schizophrenia.
